# Expression of Concern: *EARLY FLOWERING 3* interactions with *PHYTOCHROME B* and *PHOTOPERIOD1* are critical for the photoperiodic regulation of wheat heading time

**DOI:** 10.1371/journal.pgen.1011095

**Published:** 2023-12-20

**Authors:** The PLOS Genetics Editors

Following the publication of this article [1], concerns were raised regarding results presented in Fig 8. Specifically, the corresponding author informed PLOS that the underlying ChIP-qPCR expression data reported in Fig 8 were inappropriately manipulated.

The University of California, Davis confirmed that author DPW admitted to manipulation of the data underlying the results presented in Fig 8.

Fig 8 of [1] should be considered as unreliable. The authors replicated the experiment reported in this figure by re-growing the same plant materials in a growth chamber under the same conditions, performing ChIP assays at the same developmental stage, and re-analyzing the same regions of the *PPD1* promoter and controls with the same primers as reported in [1]. The authors report that they used an improved ChIP protocol [2] for the replicate chromatin immunoprecipitation analysis experiments. The protocol used for the repeat experiments is provided here:

“Kronos PS plants carrying the *elf3* mutation or a combination of this mutation and the UBI::ELF3-HA transgene were grown to the fourth-leaf stage under SD (8h light). Three grams of above-ground seedling tissue for each replicate were harvested at ZT10 and fixed in formaldehyde. Plants were harvested in the dark prior to fixation to avoid degradation of the ELF3 protein in the light. To aid in fixation penetration, harvested tissue was cut into 2–4 mm pieces. Tissue was immediately cross linked under vacuum for 20 minutes in buffer containing 400 mM sucrose, 10 mM Tris (pH 8), 1% formaldehyde, then the vacuum was turned off and the tissue sat for an additional 5 minutes. To quench the cross-linking reaction 0.25 M glycine was used and the vacuum re-applied for an additional 5 minutes. Tissue was rinsed with milli-q water three times. After fixation, tissue was frozen in liquid nitrogen and stored at −80°C until performing the chromatin immunoprecipitation as described before [95]. After extraction and sonication of chromatin, the samples were diluted to one tenth, and 10% of each sample was saved for input control. The remaining samples were pre-cleaned by rotating with 40 μL Dynabeads protein A (Invitrogen, Carlsbad) for 1 h at 4°C. After pre-cleaning, supernatants without beads were transferred to new tubes and incubated with HA-Tag (C29F4) Rabbit mAB (Cell Signaling, Danvers, MA) overnight at 4°C. The samples were then applied to freshly prepared Dynabeads protein A (Invitrogen, Carlsbad) and rotated for 2 h at 4°C. The beads were washed with 1x low-salt buffer, 1x high-salt buffer, 1x LiCl buffer and 2x TE. Chromatin elution from the beads and cross-linking reversion were performed as described in [95]. The ChIPed DNA was recovered in 80 μL of nuclease-free water by using PCR purification kit (Qiagen), and analyzed by qPCR using primers listed in Table D in S1 Text.”

S1 Fig with this notice reports the results of the replicate experiments. The underlying data for S1 Fig are in S1 File provided with this notice.

The authors stated that in the replicate experiments the enrichment for the -313 to -460 bp region in the ChIP samples from the UBI::ELF3-HA plants relative to those from the *elf3* mutant plants was similar to the results previously reported in [1], but that a stronger enrichment was observed in the -796 to -983 region. Since both the -313 to -460 and the -796 to -983 regions overlap with the deletion in the promoter of the *Ppd-A1a* photoperiod insensitive allele, the overall conclusions remain unaffected: the results of the repeat experiments support that ELF3 binds to the *PPD1* promoter and that the deletion of the binding sites in the photoperiod insensitive wheats is the likely cause of the altered expression of *PPD1* and the earlier heading under short days.

In light of these findings, the second paragraph of the ELF3 binds directly to the *PPD1* promoter *in vivo* subsection of the Results should be disregarded and the third paragraph of this subsection is updated to:

“We did not observe enrichment of ELF3 for the coding region of *PPD1* between +1514 and +1904 bp or the *FRUITFUL 2 (FUL2)* promoter (-1933 to -1750), which do not have any putative LUX binding sites and were included as negative controls (S1 Fig). The largest and most significant enrichment was detected for the -983 to -796 region in the *PPD1* promoter. We observed a marginally significant enrichment in the other promoter and 5’ UTR regions that are closer to the coding region, but we cannot rule out the possibility that those were caused by their proximity to the -983 to -796 real binding site, as the chromatin fragment sizes in this ChIP assay could be as large as 1000 bp (S1 Fig). Taken together, these experiments confirm that ELF3 acts as a direct transcriptional repressor of *PPD1 in planta* (S1 Fig), likely as part of the EC.”

The second paragraph of the Interactions between *ELF3* and *PPD1* subsection of the Discussion is updated to:

“The ELF3 repression of *PPD1* transcription is likely the result of the binding of the EC to LUX binding sites present in the region deleted in the promoter of the *Ppd-A1a* allele. This is supported by the dawn upregulation of *PPD1* in *lux* mutants in barley [31], diploid wheat *T*. *monococcum* [33, 76], and hexaploid wheat with combined mutations in all three *LUX* homoeologs [32]. LUX is a member of the EC that has a specific DNA-binding domain targeting the GATWCG motif and related promoter elements in Arabidopsis [37, 38, 77, 78]. Therefore, the similar effects of *elf3* and *lux* on the transcriptional regulation of *PPD1* suggests that the EC plays an important role in the repression of *PPD1* during the night and at dawn in the temperate grasses. In Arabidopsis, it has been shown that the EC represses the expression of *PRR7* (a close homoeolog of wheat *PPD1)*, *PRR9*, *GIGANTEA*, and *LUX* itself [37, 38, 77, 78]. Our ChIP-qPCR results confirmed that ELF3 binds to the *PPD1* promoter within the region that is deleted in the *Ppd-A1a* allele (S1 Fig), providing a possible explanation for the altered expression of *PPD-A1* in the wheat plants carrying this allele, and for their earlier heading under SD.”

The PLOS Publication Ethics team reviewed this case in collaboration with the *PLOS Genetics* Editors, and carefully considered case details including the confirmed data manipulation, that the data in question comprise a relatively minor portion of the results, and the reliability of the article’s main findings as demonstrated by the repeat data and supported by an Editorial Board member. PLOS issues this Expression of Concern to inform readers of the data manipulation concerns and that findings in Fig 8 published in [1] are unreliable. However, the journal stands by the article once amended with this notice to include these alerts and the data obtained in repeat experiments.

Mutant lines were deposited in the National Small Grains Collection under ID numbers PI 701905 (Kronos-PS, introgression of photoperiod sensitive allele *Ppd-A1b*), PI 701906 (Kronos *elf3 phyB* combined knock-outs), and PI 701907 (Kronos *elf3 ppd1*). Additional information about these accessions and/or seed requests can be done at GRIN-Global https://npgsweb.ars-grin.gov/gringlobal/search. All other relevant data are within the paper and its Supporting Information files. The underlying data and statistical analyses for S1 Figure are in S1 File (Data G) provided with this notice.

## Supporting information

S1 File Underlying data for the replicate experiments presented in the updated Fig 8 provided in S1 Fig. (XLSX)

S1 Fig Chromatin immunoprecipitation (ChIP) analysis of the *PPD1* promoter. **(A)** Gene diagram of *PPD-B1* showing the promoter regions -983 to -796, -460 to -313, -446 to -320, -142 to -21, one negative control in the *PPD-B1* coding region (+1514 to +1904) and a second negative control in the *FUL2* promoter (-1933 to -1750), all analyzed by ChIP followed by qPCR. The grey dashed line demarks the region deleted within the *Ppd-A1a* promoter present in PI. The red triangles mark the location of predicted LUX binding sites (sequences identical or similar to GATWCG [36, 37]). The *PPD1* promoter is indicated by a horizontal red line and a rectangular green box represents the first exon (ATG indicates the start codon). **(B)** Enrichment of ELF3 at four regions of the *PPD_B1* promoter and 5’ UTR in the PS*-elf3* mutant and transgenic UBI::ELF3-HA in a PS*-elf3* mutant background. No enrichment is observed in the two negative controls. Bars represent the mean ± SEM from four biological replicate experiments. ** = *P* < 0.01, * = *P* < 0.05 and ns = not significant. Primer sequences are provided in Table D in S1 Text. Raw data and statistics are in New Data G in S1 File.
(TIF)
