# Supplementary material for: EARLY FLOWERING 3 interactions with PHYTOCHROME B and PHOTOPERIOD1 are critical for the photoperiodic regulation of wheat heading time
Source: PLoS Genet. 2023 May 10;19(5):e1010655. doi: 10.1371/journal.pgen.1010655 (PMC10171656; doi:10.1371/journal.pgen.1010655)
Supplement: Attachment — Submitted filename: Answers to reviewers Alvarez Dubcovsky.pdf [file pgen.1010655.s003.pdf]

Answers to reviewers' questions

Reviewer #1

This is a very interesting and clear manuscript that demonstrates that ELF3 plays a critical role in the wheat photoperiod pathway by regulating the light signal between the phytochromes and PPD1. Loss-of-function mutations in ELF3 result in the upregulation of PPD1 at and dawn, and in early heading under both long and short days, even in the absence of PHYB. A deletion in the PPD1 promoter including an ELF3 binding region also results in earlier heading under short days, indicating that ELF3 acts as a direct transcriptional repressor of PPD1. The authors generated a large number of mutants and double mutants in different Kronos backgrounds (PI, PS). The paper is clearly written and well structured. I only have a few minor comments on the manuscript.

1A. Fig 1: It was surprising that Ppd1-A and Ppd1-B have a 4h shift in peak expression in the WT. B) Also how do you explain the very different diurnal pattern of Ppd1-B in KPI and KPS?

Authors answer: To confirm our previous results, we repeated the experiment with an independent set of RNA samples from leaves of 3-week-old Kronos PS plants collected every 4 h for 24 h. As in the original data, the *PPD-A1* peak was at ZT8 and the *PPD-B1* peak was at ZT12. The *PPD-B1* profile was slightly different, which may be due to differences in the developmental stages at which the samples were collected: 3 w for the figures below and 5 w for figure 2D-E. The results for the additional 3 w experiment are included below, but we decided not to include them in the paper since they were redundant.

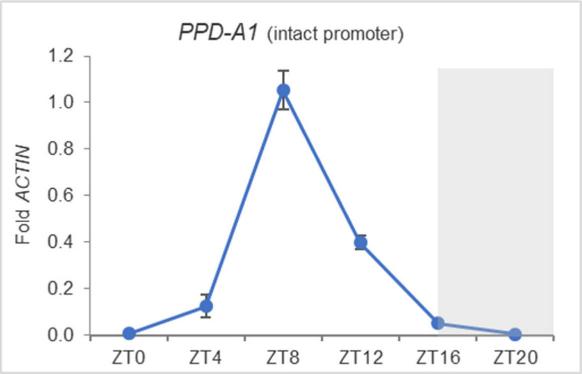

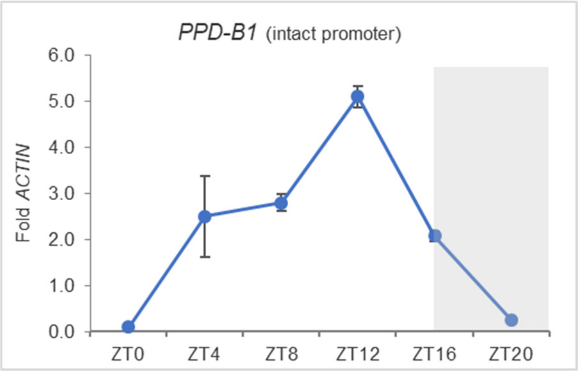

A previous study by Shaw et al. (2012, TPJ 71:71-84, Fig. 3) also showed differences in the timing of the expression peaks for *PPD-A1*, *PPD-B1,* and *PPD-D1* during the day in hexaploid wheat Paragon, suggesting that variability among homoeologs is not unusual for this gene. In the Shaw et al. 2012 study performed with Paragon under SD, the *PPD-A1* peak was at ZT6 and the *PPD-B1* and PPD-D1 at ZT3. We have also found in a previous study a 4h difference in the *PPD1* peaks between two *T. monococcum* isogenic lines differing in *ELF3* grown under LD (Alvarez et al. 2016, F.I.G. 16:365), suggesting that the precise timing of the *PPD1* peak during the day can be variable even within different accessions in the same genome.

One point to consider in the interpretation of these graphs is that the temporal resolution of these time courses is limited. Samples were collected every 4 h, so we do not know the exact time of the peak, so the differences between the *PPD-A1* and *PPD-B1* peaks might be smaller or larger than 4 h. Finally, we do not know if the differences in the timing of the expression peak during the day between homoeologs has any biological significance.

1B. Also, how do you explain the very different diurnal pattern of Ppd1-B in KPI and KPS?

Altered transcriptional profiles of the *PPD1b* alleles in the presence of a *PPD1a* allele has been reported also by Shaw et al. 2012 and by Gauley and Boden 2021. Therefore, we hypothesize that there is a feed-back regulatory loop on the *PPD1* genes, where some of the genes differentially regulated by the *Ppd-A1a* allele affect the regulation of the other *PPD1* homoeologs. We added the following sentence to make this hypothesis explicit: "The altered expression profile of *PPD-A1a* also resulted in an altered *PPD-B1* expression profile (Fig 2B and E), a phenomenon that has been previously reported for different *PPD1a* alleles in hexaploid wheat [45, 46]. These results suggest the existence of a feed-back regulatory loop where some of the genes differentially regulated by the *PPD1a* alleles affect the regulation of the other *PPD1b* homoeologs."

2. Fig 4. How do you explain that in the night break experiment (C) the second Elf3 band starts appearing only 10min after lights on, whereas in LD (Fig A) after lights on no elf3 can be seen (the gel at ZT0+10min looks different from the rest of the gel background?). Would this not suggest that not only the light but also the time of the day (circadian, or Phy night conversion) would matter for elf3 protein abundance and form? Is your idea that the lower band elf3 in the night is the active one?

Authors answer: We completely agree with reviewer 1! We have the same thought but we were afraid of complicating the story too much... We have now added the following: "However, the intensity of the upper band 10 min after the lights were turned on was very different at ZT0 than at ZT10 (Fig. 4C), suggesting that not only the light, but also the time of the day is important in the regulation of the ELF3 protein."

Regarding the second part of the question, we do think that the lower band in the night is the active one. We made that clearer in the last sentence of this section, which was modified from "These results suggest that the lower ELF3 band is critical for the repression of *PPD1*." to "These results suggest that the lower ELF3 band is the critical active form for the repression of *PPD1* during the night."

3. Since you have the elf3-HA would it not be possible to really show binding of elf3 to phys in vivo?

Authors answer: A similar question is asked by the other reviewer so we decided to test the PHYB-ELF3 interaction in wheat protoplasts using BiFC. We tested the full length PHYB and the C-terminal and N-terminal protein regions used in the Y2H experiments. All three proteins were fused with either the C-terminal (CYFP) or N-terminal (NYFP) half of the yellow fluorescent protein and tested in combinations with the respective C-terminal and N-terminal fusions of ELF3 or the YFP alone as a negative control. The protoplasts transformed with the different construct combinations were tested under light, dark, and dark with addition of the proteasome inhibitor MG132. For the light incubation we then tested three different concentrations of the two plasmids (10 µg, 5 µg, and 2.5 µg).

Unfortunately, all six PHYB constructs showed fluorescent signals when co-transformed with the other non-fluorescent half control (empty CYFP or NYFP vector) under the different conditions. Fluorescent signals were stronger under light than under dark incubation, and addition of MG132 after dark incubation did not show noticeable differences. Reducing the amounts of plasmids used in protoplast transfection resulted in weaker signals, but still

aggregated signals were observed in the negative control using one of the non-fluorescent YFP halves. The signals were concentrated in few aggregates outside the nucleus (see figure below). Although the CYFP-C-PHYB combined with the NYFP-ELF3 showed a stronger signal than when combined with the NYFP alone (under light, figure below), we do not feel confident enough to include this result in the paper.

CYFP-C-PHYB co-transformed with NYFP

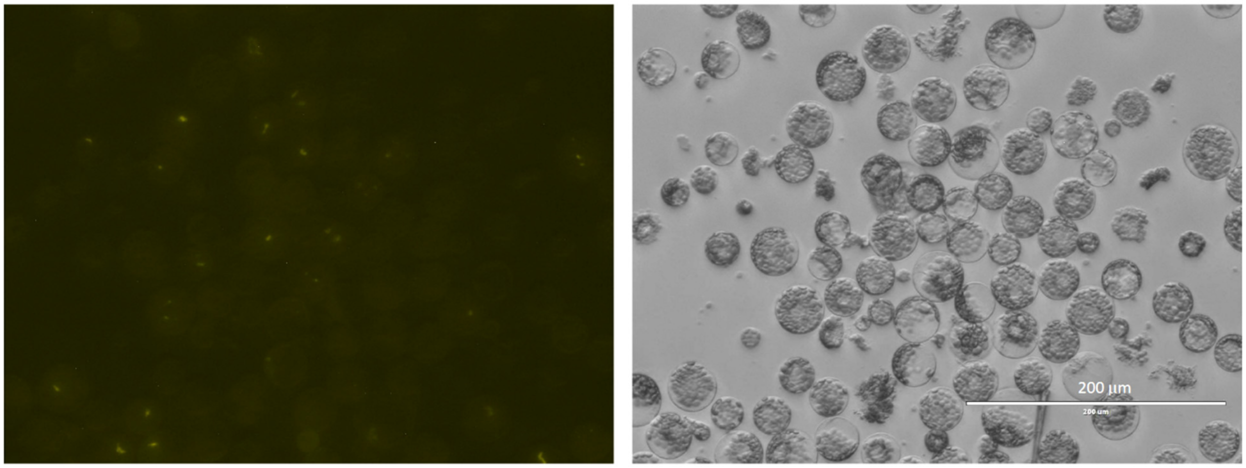

CYFP-C-PHYB co-transformed with NYFP-ELF3

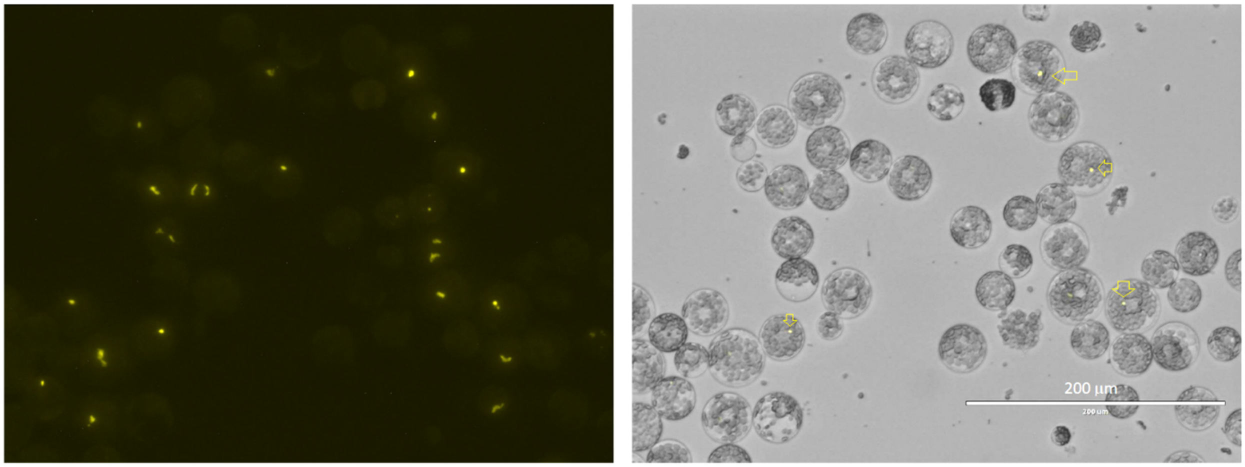

It seems that testing this interaction will require a lot of additional work, which will be better done in a follow-up study. We edited the sentence in the discussion to make explicit that the *in planta* validation of these results in wheat is still pending: 'The genetic interactions between *ELF3* and the phytochromes were also reflected in a physical interaction between the ELF3 protein and both PHYB and PHYC proteins in Y2H assays (Fig 3). Although these interactions still need to be validated *in planta* for wheat, *in planta* interactions between phytochromes and all three members of the EC have been reported before in Arabidopsis, where they have been proposed to be important in the connection between the circadian clock and the light signaling pathways [65, 66].'

4. Fig. 5 Please, explain in the M&M part what exactly is ppd1-null is and how this line was generated.

Authors answer: Sorry, we corrected *ppd1-null* in Fig 5 to *ppd1*, which is described in M&M as a mutant described before in Pearce et al. (2017) and in more detail in S1C Fig as "The *ppd1* line carries a premature stop codon in *ppd-A1* that eliminates 514 amino acids of the *PPD-A1* protein, including the highly conserved CCT domain, and a gamma ray-induced deletion in *ppd-B1* that eliminates the complete gene (Pearce et al., 2017; Shaw et al., 2020).".

5. On page 17 you write: The notable exception is the downregulation of VRN2, CO1, and CO2 at dawn in elf3 phyB (S3D, F, and H) relative to elf3 (Fig7G, J and M), suggesting the PHYB is required for the upregulation of these genes in the elf3 mutant background at dawn.

6. In S3D VRN is upregulated in elf3 phyb, actually in all mutants VRN2 is upregulated, also in the phyb mutant and this is somewhat puzzling. It might also be indirect effects through feedback loops between flowering time genes (Ppd1, VRN1, CO, VRN2, FT1) as is actually indicated in your flowering model.

Authors answer: It was difficult to organize the large amount of expression data and we prioritized making clear the most critical comparisons in graphs including only two curves. One thing we sacrificed, was the ability to compare the WT, *elf3* and *elf3 phyB* in the same graph, requiring a comparison across different figures.

We focused on ZT0 because *PPD1* expression showed that this is a critical time point for the differences between PI and PS wheat, and we think is critical for the photoperiodic response. At ZT0, *VRN2* is not significantly upregulated in the *phyB* (S3C) or the *elf3 phyB* (S3D Fig) but is significantly upregulated in *elf3*. In other time points (ZT16 and ZT20), *VRN2* is upregulated also in the *phyB* mutant, as pointed by the reviewer.

We also agree with the reviewer, that the requirement of *PHYB* for the upregulation of these genes in the *elf3* mutant background can be in many cases an indirect effect of the complex regulatory feedback loops among these genes. We rephrased this paragraph to incorporate this suggestion from the reviewer: "It is interesting to point out that at dawn (ZT0), *PPD1*, *VRN1*, *VRN2*, *CO1*, and *CO2* are expressed at lower levels in *elf3 phyB* (Fig 2 and S3) than in *elf3* (Fig 6 and 7), suggesting that at this time point *PHYB* is required for the upregulation of these genes in the *elf3* mutant background. However, given the complex feedback regulatory loops that exist among these genes, we do not know which of these effects are direct or indirect.".

7. As the authors provide a model for a barley flowering time pathway with ELF3 in a central position, but also discuss the possible role of LUX, it might be nice to add LUX to the model in Fig. 9

Authors answer: We modified the model in Figure 9 to include LUX and ELF4 as part of the Evening complex, and added to the legend: "ELF3, LUX and ELF4 proteins form the evening complex, which binds to the *PPD1* promoter and inhibits its transcription."

Reviewer #2

The authors provide a concise genetic analysis of photoperiodic flowering in the temperate cereal wheat. Through the phenotypic and molecular analysis of single and double mutants they show that ELF3 acts downstream of PHYB in the induction of the flowering activator PPD1, with ELF3 being a negative regulator of PPD1 expression. They further show that PHYB does not affect ELF3 gene expression but rather interacts with the PHYB photoreceptor

(and PHYC) (in yeast two-hybrid), suggesting that PHYB regulates ELF3 activity, possibly by light-mediated post-translational modification of the ELF3 protein. They further show by ChIP that ELF3 directly binds the promoter of PPD1.This is a very thoughtful study. The data support the conclusions drawn. The manuscript is well-written. This manuscript stands out because it not only genetically analyzes ELF3 function but also addresses a possible mechanism of ELF3 regulation by studying the ELF3 protein. I only have a few minor comments:

1. Fig. 4A. In this Western blot, the ELF3-HA protein runs at about 130 kDa. However, ELF3 is predicted to be only approx. 75 kDa in size. That is quite a discrepancy. I ask the authors to show a control blot with a protein extract of a non-transgenic plant as a control in order to confirm that the detected band(s) are indeed ELF3-HA. In the supplement, please also show a complete blot including the region of the gel where 75 kDa proteins are expected.

Authors answer: We agree with Reviewer # 2 that the ELF3-HA band in the western blots is higher than the expected 88.5 KDa (ELF3 plus the 3xHA tag). However, it is not 130 kDa but closer to 105-110 kDa. We generated additional blots with both the pre-stained protein marker PagerulerTM Prestained Protein Ladder (ThermoFisher) and the unstained protein maker (PagerulerTM Unstained Protein Ladder, ThermoFisher) to get more accurate estimates of the band sizes. Based on these markers, the size of the ELF3-HA band was estimated to be ~110 kDa. We do not know the cause of this discrepancy but a similar difference was observed in rice blots detected with an ELF3 antibody (Andrade et al. 2020). We added a supplemental S6 Fig including the additional size markers and also non-transgenic plants as negative controls, as requested by the reviewer. No band was detected in the negative controls confirming that the ~110 kDa band was the ELF3-HA (S6A Fig). We also added a complete blot to show the absence of other bands (except rubisco) as requested by the reviewer (S6A Fig). We added the following sentence in the text: "The ELF3-HA protein detected by immunoblotting using an anti-HA antibody was approximately 110 kDa, which is slightly higher than the predicted 88.5 kDa both in protein extracted from UBI::ELF3-HA transgenic plants and from transformed protoplasts (S6A-C Fig). This was the only band detected in the blots (except for rubisco) and it disappeared in the non-transgenic controls confirming that it is ELF3-HA. A higher than expected size for the ELF3 protein was also observed in western blots in rice [39]."

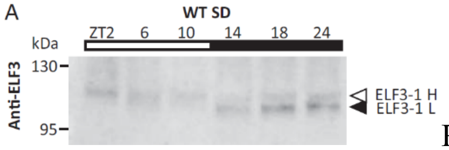

Rice result from Andrade et al. 2022

2. I wonder if the authors have confirmed the PHYB-ELF3 interaction using additional methods? E.g. split luciferase, BiFC. This would strengthen the manuscript. However, since this interaction is described for Arabidopsis, I do not require these experiments.

Authors answer: see answer to reviewer #1.

3. Fig. 4: The authors speculate that the upper ELF3 band might reflect PIF3 phosphorylation or ELF3 ubiquitination. A treatment of the extract with phosphatase could easily test for phosphorylation and would strengthen the manuscript.

Authors answer: In the native state the ELF3 protein is unstable which make its purification or the Co-IP experiments very difficult. We have attempted the Co-IP without success so far. Therefore, we have not been able to perform the suggested phosphorylation experiment.
