# Supplementary figures and images for: Expression of Concern: EARLY FLOWERING 3 interactions with PHYTOCHROME B and PHOTOPERIOD1 are critical for the photoperiodic regulation of wheat heading time
Source: PLoS Genet. 2023 Dec 20;19(12):e1011095. doi: 10.1371/journal.pgen.1011095 (PMC10732402; doi:10.1371/journal.pgen.1011095)

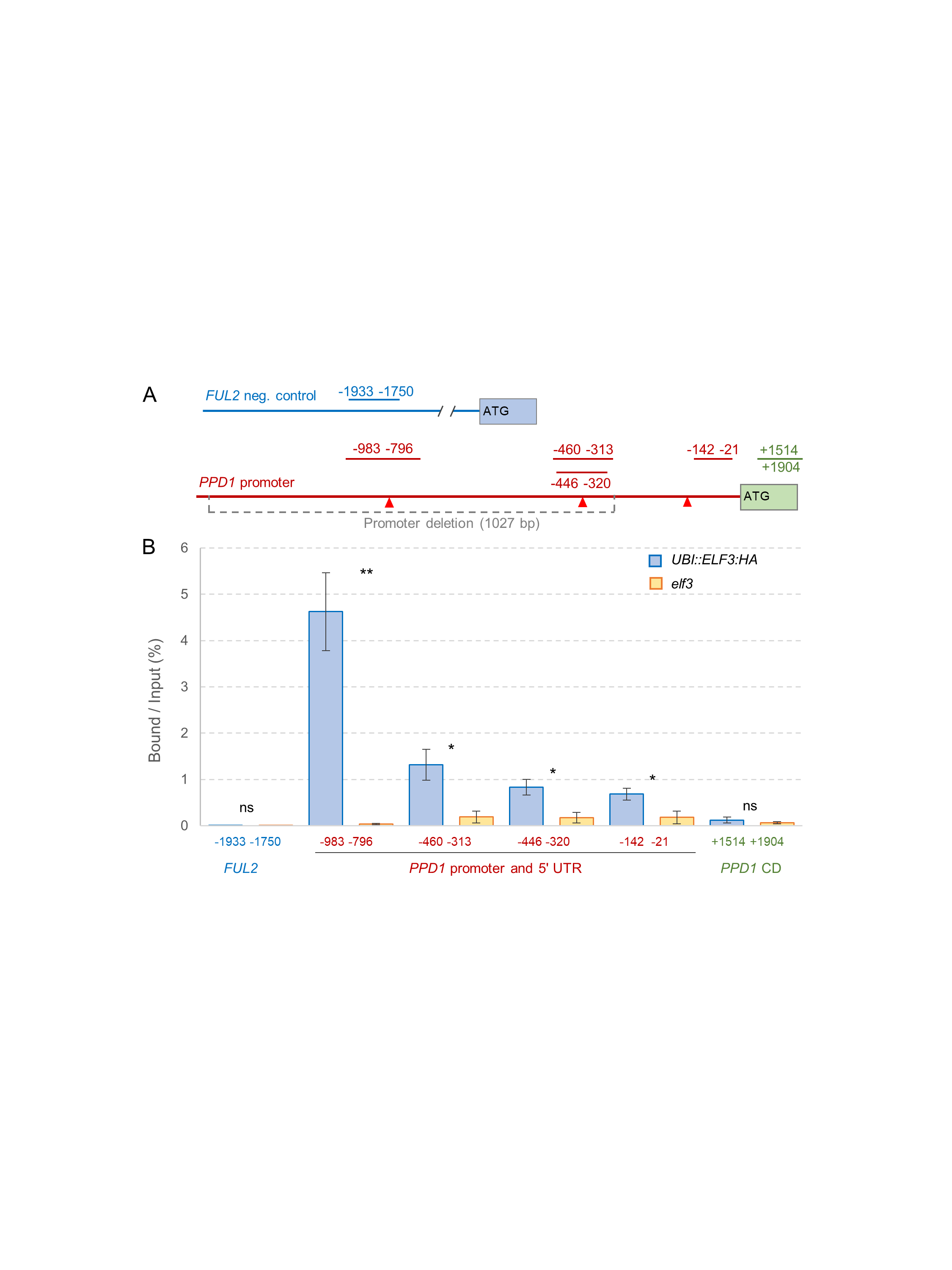

Supplement: S1 Fig — (A) Gene diagram of PPD-B1 showing the promoter regions -983 to -796, -460 to -313, -446 to -320, -142 to -21, one negative control in the PPD-B1 coding region (+1514 to +1904) and a second negative control in the FUL2 promoter (-1933 to -1750), all analyzed by ChIP followed by qPCR. The grey dashed line demarks the region deleted within the Ppd-A1a promoter present in PI. The red triangles mark the location of predicted LUX binding sites (sequences identical or similar to GATWCG [36, 37]). The PPD1 promoter is indicated by a horizontal red line and a rectangular green box represents the first exon (ATG indicates the start codon). (B) Enrichment of ELF3 at four regions of the PPD_B1 promoter and 5’ UTR in the PS-elf3 mutant and transgenic UBI::ELF3-HA in a PS-elf3 mutant background. No enrichment is observed in the two negative controls. Bars represent the mean ± SEM from four biological replicate experiments. ** = P < 0.01, * = P < 0.05 and ns = not significant. Primer sequences are provided in Table D in S1 Text. Raw data and statistics are in New Data G in S1 File. (TIF) [file pgen.1011095.s002.tif]
